# Unusual Presentation of Leiomyoma in the Hindfoot

**DOI:** 10.1155/2024/1217277

**Published:** 2024-03-14

**Authors:** Abdullah Zaher, Jaouad Yasser, Daniel Badaro, Noureddine Sekkach

**Affiliations:** ^1^Department of Orthopedics and Traumatology, Centre Hospitalier de Saint-Denis, Hôpital Delafontaine, Saint-Denis, France; ^2^Centre de Pathologie Bichat, Paris, France

## Abstract

A leiomyoma is a benign smooth muscle tumor that is most commonly found in the uterus. Limited studies have reported leiomyoma of the foot, rendering it an usual finding. We present a case of a 57-year-old female patient who presented to the clinic for a painless mass on the posteromedial side of the right heel. It was diagnosed by the radiologist on MRI as a probable schwannoma. The patient underwent surgical excision of this mass which turned out to be a leiomyoma on pathology report. Although foot leiomyoma is a rare finding, leiomyoma should be listed in the differential diagnosis when evaluating foot soft tissue masses. It is difficult to confirm the diagnosis clinically and radiographically, yet a histologic evaluation can affirm the diagnosis. Surgical excision is the treatment of choice offering immediate symptomatic relief.

## 1. Introduction

A leiomyoma is a benign smooth muscle neoplasm that is primarily found in the uterus. It can affect 80% of women [1]. However, it can arise in other locations where there is smooth muscle such as in the gastrointestinal tract [2]. There is little evidence in the literature of cases of leiomyomas at the level of the extremities, with higher incidence in the lower extremities compared to the upper extremities.

Since leiomyomas can occur at varying depths, their classification can be based on where they are found. Cutaneous leiomyomas originating from the arrector pili muscle are the most superficial. Dermal vascular smooth muscle is the origin of subcutaneous leiomyomas. Last but not least, profound leiomyomas are incredibly uncommon in the extremities [3].

When a patient presents to the clinic with a foot mass, a list of differential diagnosis can be put without including the case of leiomyoma as it is a veritably rare diagnosis. Even though a leiomyoma's growth is benign, it can have a painful mass compressive impact. This case report describes the rare finding in a female patient who had a leiomyoma on the posterior aspect of the heel.

## 2. Case Report

A 57 year old female—working as a director in a nursery—without any medical history presented to the orthopedics clinic for the first time with the chief complaint of the presence of a small mass at the level of the right heel. The mass was noticed 1 year prior to presentation, but it gained in size that it started annoying her while putting on her shoes.

The mass is raised, firm, and localized slightly medial to the posterior aspect of the right heel.

The patient does not practice any kind of sports, and she did not have any history of heel injuries or wounds, constitutional symptoms, or weight loss.

During the physical examination, a small noncompressible lump measuring 1 by 1 centimeter was discovered slightly medial to the posterior side of the right heel. Deep palpation revealed a slightly sensitive lump. Distal neurovascular examination results were unremarkable. There was no concomitant tingling, numbness, redness, or heat.

On presentation, the patient presented the results of an ankle MRI—realized outside our hospital—ordered by her general physician. The radiologist described the finding of a superficial nodule in contact with the posterior part of the Achilles tendon measuring 9 × 9 × 6 mm that is vascularized (gadolinium-enhanced zone). The radiologist's most probable diagnosis was a schwannoma.

**Figure 1:**
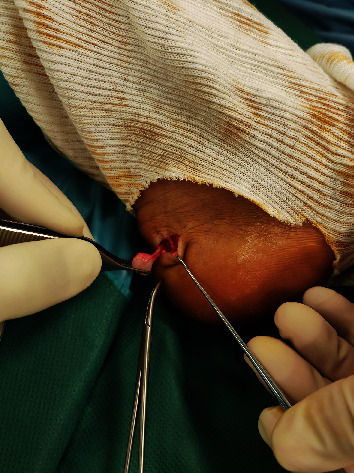
Excision of tumor intraoperatively.

Two weeks later, the patient was due for surgery. After being put to lie down in a supine position, the patient was given locoregional anesthetic. A calf tourniquet was inflated, after which a small incision was made over the tumor. It was discovered that the tumor was superficial and was attached to the soft tissues beneath it. The mass was a whitish nodule, about 1 × 1 × 1 cm in size (Figure 1). After the mass was removed, it was submitted for anatomopathological analysis.

**Figure 2:**
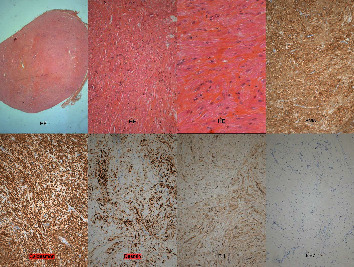
Histopathological sections of the excised tumor. HE: hematoxylin-eosin; SMA: smooth muscle actin; FH: fumarate hydratase.

The pathology report described a regular fusiform cellular proliferation without nuclear atypia that is compatible with leiomyoma. Immunohistochemistry showed the diffuse expression of anti-actine smooth muscle antibodies, caldesmon and desmin, in the absence of tumor markers. The reports conclude with the diagnosis of leiomyoma without histological signs of malignancy (Figure 2).

One- and four-month postoperative follow-up visits showed a healed incision without any signs of infection, or mass remnants, or recurrence.

Due to the unusual diagnosis, the patient was asked to do a check-up with her gynaecologist, which turned out to be insignificant for any abnormal findings.

She attests to the lack of any personal or family history of skin cancer, renal cell carcinoma, uterine tumors, or soft tissue masses.

## 3. Discussion

Tumors are still an uncommon cause of heel masses while making a differential diagnosis. The foot and ankle are home to just 8% of benign soft tissue tumors, which include leiomyoma, giant cell tumors, and lipomas. While the foot and ankle account for 5% of all malignant soft tissue masses, Kaposi sarcoma, clear cell sarcoma, and malignant fibrous histiocytoma are the most common types of these tumors [4].

A leiomyoma is by definition a benign smooth muscle tumor. It mostly affects the uterus, and it is quite uncommon for it to appear in the foot or ankle.

Upon reviewing the literature, it was found that there were not many published cases of foot leiomyoma. Examples include the case series of Szolomayer et al., which included eight patients with excised leiomyomas of the ankle, plantar foot, and hallux [5], and Jalgaonkar et al., on the other hand, which described a case of a leiomyoma in the plantar side of the forefoot that was primarily identified as a fibroma [6]; Stock et al.'s case of leiomyoma in the dorsolateral aspect of the foot of a 50-year-old male patient, where it was a slowly growing mass over 10 years before undergoing core needle biopsy without excision [7]; Savage et al.'s 2019 report of a patient with a plantar arch leiomyoma [8]; and Buddemeyer et al.'s 2018 case report detailing the discovery of a 41-year-old female patient's cutaneous leiomyoma in the heel [9].

On rare occasions, these benign leiomyomas may progress to malignant leiomyosarcomas. According to De Vos et al., benign leiomyoma to malignant leiomyosarcoma transforms between 0.13% and 0.29% of the time [10]. Moreover, for the time being, there exist no reported cases of foot or ankle leiomyosarcoma.

On another note, Reed's syndrome, which is brought on by an inactivating mutation of the tumor suppressor fumarate hydratase and results in hereditary leiomyomatosis and renal cell carcinoma, may be linked to the presence of superficial leiomyomas [11].

In addition to uterine leiomyomas in women, this condition can cause renal cell carcinomas and cutaneous leiomyomas.

Therefore, cutaneous leiomyomas should raise a clinician's concern of this disease, and if a patient meets the diagnostic requirements, genetic testing ought to be carried out [12]. For this condition, there exist major and minor criteria; the major one is the finding of multiple cutaneous leiomyomas with at least one positive biopsy, whereas the minor criteria include the finding of a solitary cutaneous leiomyoma, family history of hereditary leiomyomatosis, early-onset renal papillary tumor, and multiple early-onset symptomatic uterine fibroids.

In our case, the patient presented with a solitary nodule of the heel turning out to be a cutaneous leiomyoma, which is one of the minor criteria, yet the other criteria do not apply to our patient.

Concerning the management of foot leiomyoma, as demonstrated in our case, the identification of leiomyoma offers a positive prognosis. The mainstay of the therapeutic management for a solitary mass is the surgical excision which offers immediate symptom relief. Besides, the surgical excision was essential in confirming the diagnosis of this mass.

In conclusion, even though it is extremely uncommon to discover a foot leiomyoma, the foot and ankle expert should always consider the leiomyoma as a possible diagnosis when addressing a soft tissue mass. Foot leiomyomas are difficult to diagnose clinically and paraclinically; therefore, the diagnosis can only be established by a histologic examination. The preferred course of treatment for this condition is excision.
